# Perspectives of health workers on the facilitators and barriers to antiretroviral therapy adherence following intensive adherence counseling in Northern Uganda

**DOI:** 10.3389/frhs.2025.1387823

**Published:** 2025-01-28

**Authors:** Humphrey Beja, Daisy Nakayiwa, Innocent Ocitti Owachgiu, Micheal Tonny Edek, Veronic Kobusinge, Oscar Akaki, Samson Udho

**Affiliations:** ^1^ Department of Midwifery, Faculty of Nursing and Midwifery, Lira University, Lira, Uganda; ^2^ Department of Psychiatry, Faculty of Medicine, Lira University, Lira, Uganda; ^3^ Department of Community Health, Faculty of Public Health, Lira University, Lira, Uganda

**Keywords:** facilitators, barriers, antiretroviral therapy, intensive adherence counselling, healthcare-workers

## Abstract

**Background:**

In some contexts, people living with HIV (PLWH) who are virally non-suppressed and participating in an intensive adherence counseling (IAC) program have demonstrated non-adherence to antiretroviral therapy (ART) even after IAC. There is limited literature on the facilitators and barriers to ART adherence following IAC.

**Objective:**

This study aimed to explore the perspectives of healthcare workers (HCWs) on the facilitators and barriers to ART adherence following IAC among PLWH in Northern Uganda.

**Methods:**

This was a descriptive qualitative study conducted among HCWs at the ART clinics of the two highest-volume public health facilities in Lira District. We purposively sampled 15 study participants and conducted face-to-face in-depth interviews using an interview guide formulated based on the components of the Capability, Opportunity, and Motivation framework for Behavior change (COM-B framework). Thematic analysis was used based on the COM-B framework. In this study, the desired behavior was ART adherence following IAC. Factors that were perceived to positively affect any component of the COM-B framework were classified as facilitators and those that were perceived to negatively affect were classified as barriers.

**Results:**

The majority of the participants were females (53%), diploma holders (40%), and nurses (40%). The perceived facilitators and barriers to ART adherence following IAC emerged as six key themes under the subdivisions of the three domains of the COM-B framework: cognitive and emotional processes, physical and practical skills, accessibility and material resources, social relationships and cultural dynamics, cognitive beliefs and aspirations, and finally, *emotional* and subconscious drivers. These themes were identified as either facilitators or barriers to ART adherence following IAC depending on the lenses of interpretation.

**Conclusions:**

This study offers a multidimensional insight into the facilitators and barriers to ART adherence following IAC and how the behavior influencing ART adherence can be optimized. The results suggest that optimizing cognitive and emotional processes, physical and practical skills, accessibility and material resources, social relationships and cultural dynamics, cognitive beliefs and aspirations, and emotional and subconscious drivers during IAC and any ART adherence-related intervention could yield the best level of ART adherence among the PLWH who are virally non-suppressed and on ART.

## 1 Background

The morbidity and mortality associated with HIV/AIDS for years have remained alarming, with the disease still incurable despite current developments in drugs and therapeutics. Globally, the number of people living with HIV/AIDs (PLWH) is estimated to be approximately 38.4 million. The most affected region is sub-Saharan Africa, with approximately 25.6 million PLWH, 730,000 new infections, and 300,000 AIDS-related deaths (1). The number of PLWH in Uganda is approximately 1.4 million, and AIDS-related deaths stand at 22,000 (2). In Uganda, 5.6% of people aged 15–49 years are living with HIV with the majority being females (6.9%) compared to males (5.3%) (2).

Testing for HIV and subsequent enrollment of those found to be HIV-positive into antiretroviral therapy (ART) are the cornerstones of HIV viral load suppression. Viral load suppression is said to be achieved by PLWH when the viral load is below 1,000 copies/ml of blood (3). Globally, 85% of PLWH were aware of their HIV status, 75% were on ART, and 68% had a viral load below 1,000 copies/ml of blood in 2021 (4). In Uganda, 91% of the PLWH were aware of their HIV status, 90% were on ART, and 82% had a viral load below 1,000 copies/ml of blood in 2021 (5). At global, regional, and national levels, the target for HIV viral load suppression has lagged behind HIV testing and treatment enrollment (4, 5). Specifically, the goal of the Joint United Nations Program on HIV/AIDS (UNAIDS) to achieve 90% viral load suppression for all PLWH by the end of 2020 was not met (5).

In 2019, the Uganda AIDS Control Program (ACP) prioritized three strategies to accelerate the attainment of a viral load of less than 1,000 copies/ml of blood among people living with HIV (PLWH). These strategies included a specialized form of counseling called intensive adherence counseling (IAC), repeating the viral load test, and switching to an optimal ART regimen in cases of non-response or drug resistance. Among these, IAC is arguably the most difficult strategy to implement (6). IAC is a systematic and rigorous way of counseling that can be summarized using the 5A framework (3). The 5A's include the following: (1) *assess*—explore the reasons for non-suppressed viral load; (ii) *advise*—share the advantages of adhering to the treatment and the anticipated outcomes; (iii) *assist*—evaluate some of the suspected hindrances to treatment adherence and jointly brainstorm strategies to overcome them; (iv) *agree* with the client on how to deal with the non-suppressed viral load based on the identified barriers; and (5) *arrange* the return date for ART medication refill and the date for the next IAC and request patient to bring evidence of adherence to the agreed-upon plan (3).

IAC has yielded mixed findings in Uganda with some studies showing that IAC results in improved viral load suppression among PLWH who had non-suppressed viral load (7, 8) while some studies found contrary results (9, 10). Subsequently, studies have explored HIV clients' and caregivers' perspectives of enhancers and hindrances to viral load suppression following IAC (11, 12). However, the perspectives of HIV/AIDS healthcare workers (HCWs) are not well documented to fully understand facilitators and barriers to ART adherence following IAC in Uganda. Using the Capability, Opportunity, and Motivation framework for Behavior change (COM-B framework), this study aimed to explore healthcare workers' perspectives on the facilitators and barriers to ART adherence following IAC among PLWH seeking care in government-owned healthcare facilities in Northern Uganda. This study may produce results that may be useful in the design of innovative approaches that may result in the realization of the optimum outcome of IAC.

### 1.1 The COM-B theoretical framework

We adapted the COM-B theoretical framework for behavior change to assess the facilitators and barriers to ART adherence following IAC. This model proposes that the performance of a behavior is premised on one's psychological, physical, environmental, and social opportunities available for one to participate in a given behavior, and the personal desire to carry out the behavior in preference to other competing behaviors (13, 14). This framework consists of four key components, which are the capability (C), opportunity (O), motivation (M), and the desired behavior (B) (COM-B framework) (13, 14). The abilities, knowledge, and skills to adhere to ART following IAC form the capability component in this study (13, 14). The external factors that influence the ability of an individual to adhere to ART following IAC form the opportunity component in this study. On the other hand, the influences that lie within an individual that guide decision-making for ART adherence to achieve viral load suppression form the motivation component in this study (13, 14).

**Figure 1:**
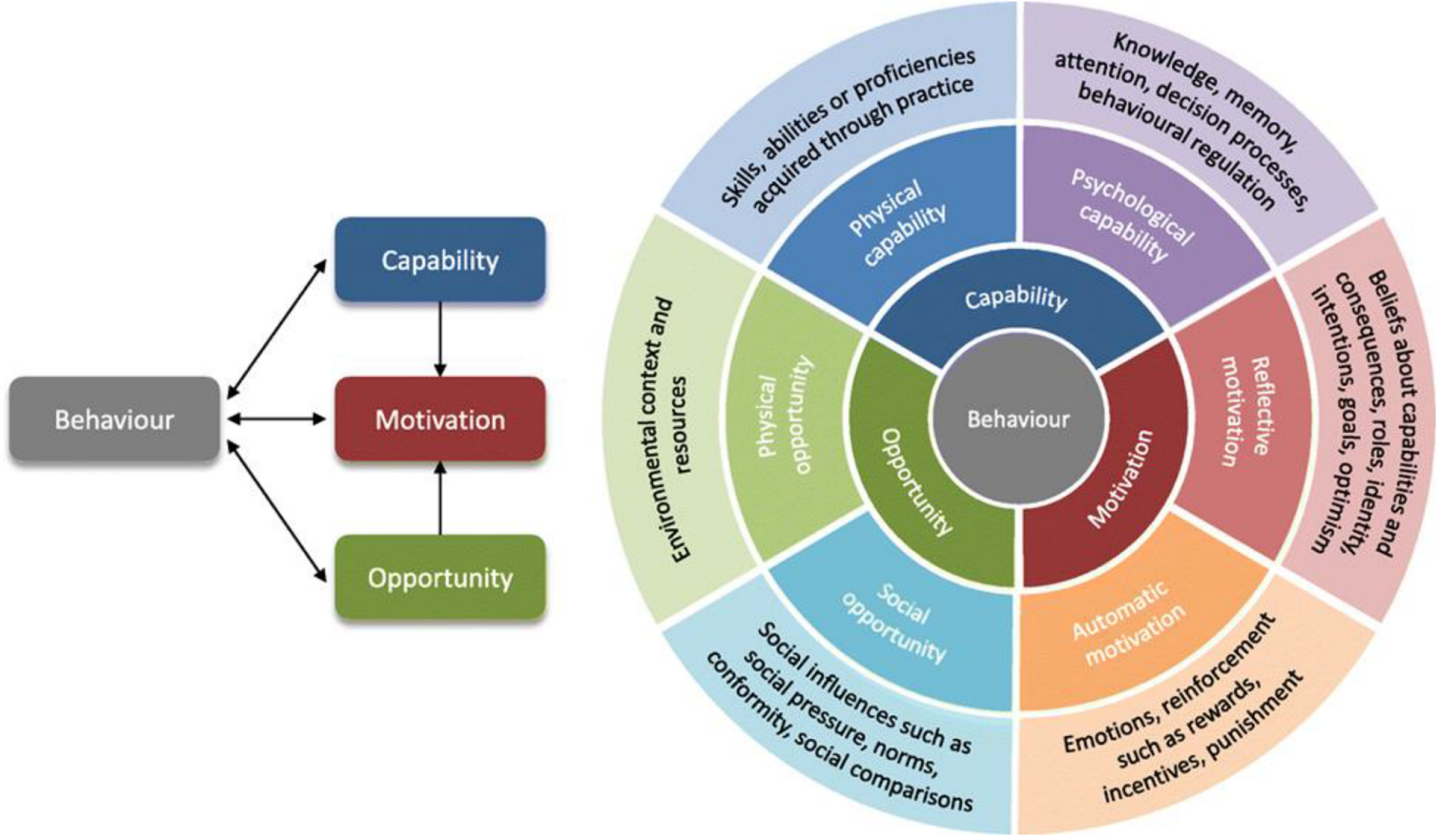
COM-B theoretical framework for behavior change.

The COM-B model was chosen for this study not only because of how the framework perfectly fits into the study context but also because of its comprehensive and adaptable approach to understanding behavior change, identifying barriers and facilitators underlying a particular behavior that represent potential targets for future intervention design (13, 14). The model is focused on three key factors: capability, opportunity, and motivation; provides a holistic framework for assessing the complex, multidimensional nature of ART adherence following IAC since each of these factors directly influences the behavior of individuals; and allows for an in-depth exploration of these influences in a structured manner (13, 14). Additionally, COM-B's flexibility in application across various settings and its integration with behavior change techniques (such as interventions tailored to specific needs) made it particularly suitable for our study in the rural context of northern Uganda (13, 14). While other frameworks like the Health Belief Model (15, 16) or the Theory of Planned Behavior (17) also have merit, COM-B was more fitting due to its capacity to address not only individual-level factors but also environmental and social influences on behavior (18). These other frameworks will be used during the discussion of the results of this study. This aligns with our focus on understanding the diverse facilitators and barriers to ART adherence following intensive adherence counseling in this setting. The COM-B framework is shown in Figure 1.

## 2 Materials and methods

### 2.1 Study design and setting

This was a descriptive study employing a qualitative approach to data collection conducted in May 2022 in rural Uganda to explore the perspectives of healthcare workers on the facilitators and barriers to ART adherence following IAC. The study was conducted in Lira District, Northern Uganda, specifically at the ART clinics of the level IV health centers (HC) of Ogur and Amach. Lira District is about 342 km by road to the capital city of Uganda (Kampala). In Uganda, the healthcare system is structured into levels based on the population served and service complexity (19). Health Center I relies on village health teams (VHTs) for preventive care and referrals, while Health Center II offers outpatient services for common illnesses and basic maternal care. Health Center III includes inpatient care, maternity services, and basic laboratory diagnostics (19). Health Center IV, serving as a mini-hospital, provides more advanced care, including surgical services, and acts as a referral center for lower-level facilities (19). Beyond HC IV, district hospitals offer specialized care and cater to broader populations, while regional and national referral hospitals provide highly specialized services, including training and research facilities. This hierarchical structure ensures an efficient referral system and equitable access to healthcare across Uganda. The Uganda health system permits ART services, including intensive adherence counseling (IAC) for individuals with non-suppressed viral loads to be offered at Health Center III and above (19). Our study district had only two Health Center IV facilities and five Health Center III facilities. Each of the two Health Center IV facilities has a functional ART clinic that operates from 8:00 a.m. to 5:00 p.m. on weekdays. The ART clinics offer IAC services at least once a week. In each of the ART clinics, there are auxiliary staff, at least two counselors, two nurses, and one clinical officer. This study selected Health Centre IV facilities because their catchment areas include populations served by Health Centre III facilities, enhancing the generalizability of findings.

### 2.2 Population and sample size

We conducted this study among healthcare workers at the ART clinics of Health Center IV in Ogur and Amach, Northern Uganda. A purposive sampling technique was used to recruit 15 participants who met the inclusion criteria (20). The information power principle was used in sample size estimation where after each interview, the audio-recorded interviews were transcribed, and the researchers and interviewers reviewed the transcripts and discussed whether new significant information was still emerging. These discussions helped assess whether additional interviews were necessary. By the 15th interview, no new themes or significant insights emerged, indicating that data saturation had been achieved. Consequently, we concluded that 15 participants were sufficient to meet the study objectives (21).

### 2.3 Sampling criteria

The study included healthcare workers directly involved in delivering intensive adherence counseling (IAC) services to PLWH at selected Health Center IV facilities. To ensure participants had sufficient familiarity with IAC and its implementation challenges, only those with a minimum of 6 months of experience providing IAC services were eligible. This criterion was chosen to capture perspectives from individuals with a robust understanding of the facilitators and barriers to ART adherence following IAC. Healthcare workers in administrative roles without direct involvement in IAC or those with less than 6 months of IAC experience were excluded from the study. Healthcare workers with busy clinical schedules and those unwilling to participate were initially planned for exclusion, but none of the approached participants declined due to these reasons, so this did not impact our study’s findings.

### 2.4 Recruitment of participants

During recruitment of the study participants, the person in charge of the ART clinic worked with trained data collectors to determine the eligibility of potential study participants at each health facility. Once potential study participants were identified, the data collectors discussed the purpose of the study, its importance, and any possible risks associated with participation in the study. The potential study participants were given a chance to choose whether or not they participate in the study. Those who were eligible for inclusion and were agreed to participate were asked to sign an informed consent form. The data collector and the study subject then agreed on a mutually convenient time and location for the in-depth interview.

### 2.5 Data collection method, tools, and procedures

This study involved face-to-face in-depth interviews (IDI) conducted using a semi-structured interview guide with open-ended questions, which were formed based on the COM-B theoretical framework for behavior change. The questions were tailored to explore healthcare workers’ perspectives on the facilitators and the barriers to ART adherence following IAC. The IDI guide underwent pilot testing with healthcare workers, who were not included in the study. After the pilot testing, the data collection tool was revised as needed. The IDI guide consisted of five main sections: the four components of the COM-B theoretical framework and the sociodemographic characteristics of the study participants. The data collectors posed open-ended questions to the study participants to bring out unbiased information within each section of the data collection tool (Supplementary Appendix 1). The study participants were interviewed either in a confidential area at the health center or in their homes. All interviews were conducted in English, each lasting approximately 60 min, and were recorded using an audio recorder.

### 2.6 Data management and data analysis

The collected data were transcribed *verbatim* by the data collectors. They verified the transcript by listening to the audio recording and comparing it with the transcript to ensure that no important information was left out and that the interviews were accurately transcribed before proceeding with the next interview. After each interview, the researchers reviewed the data, reflected on emerging codes, and adjusted the approach for subsequent interviews as necessary. This process continued until no new significant information was identified, allowing the researchers to refine the data collection process based on ongoing insights. The coding process began with an inductive approach, allowing themes to emerge naturally from the participants' narratives. During this phase, the data were segmented into meaningful units and labeled with descriptive codes that reflected the content of the responses. Once the initial codes were developed, a deductive process was used to map them onto the three components of the COM-B model: capability, opportunity, and motivation. This combined inductive and deductive approach ensured that the themes were grounded in the participants' experiences while remaining aligned with the theoretical framework (22). We used a thematic analysis technique to code and allocate the codes under each domain, and a code book was used to ensure consistency. After allocating the codes to each domain of the COM-B model, similar codes under each domain were grouped under each of the two subdivisions of the COM-B model, and high-level themes were generated. The research team then had a discussion and resolved any inconsistencies, and they agreed on the high-level themes presented in the Results section of this paper. All the authors came to a consensus on the final themes presented as results.

### 2.7 Rigor and trustworthiness of the study

In maintaining trustworthiness and rigor in this study, the dependability, credibility, transferability, and confirmability principles were employed (23). Credibility was achieved through purposive sampling, which selected participants with relevant experience in intensive adherence counseling (IAC) for people living with HIV (PLWH). This ensured the data collected were accurate and reflective of the participants' true experiences. Additionally, member checking was performed, where some participants were asked to review and confirm the findings, ensuring that their interpretations aligned with their perspectives (24). Dependability was addressed by maintaining a detailed audit trail throughout the study, documenting each step of the research process, from data collection to analysis, ensuring that the process could be followed and replicated by others (24). For transferability, comprehensive descriptions of the study context, participants, and methodology were provided, enabling others to assess whether the findings are applicable in different settings (24). Lastly, confirmability was ensured by the researchers maintaining neutrality during data collection and analysis and by using data triangulation, which involved gathering information from multiple sources (healthcare workers in different roles and facilities, observation field notes, and interview transcripts) (25). This helped ensure that the findings were based on the data rather than influenced by researcher bias, thereby enhancing the study's objectivity (24). Together, these strategies contributed to the rigor and trustworthiness of the study, ensuring that the findings are credible, reliable, applicable, and unbiased (24).

## 3 Results

### 3.1 Sociodemographic characteristics of the study participants

A total of 15 in-depth interviews were conducted with healthcare workers at Ogur and Amach Health Center IV. The study participants were aged 20–50 years; most of them were females (53%), diploma holders (40%), and nurses (40%) Table 1.

**Table 1:** Demographics of the study participants.

Study participants (S)	Age	Sex	Education level	Carder
S 1	24	F	Degree	Counselor
S 2	33	M	Completed lower secondary	Community linkage facilitator
S 3	40	M	Completed lower secondary	Village health team member
S 4	33	M	Completed lower secondary	Community linkage facilitator
S 5	50	M	Diploma	Data clerk
S 6	44	F	Diploma	Assistant nursing officer
S 7	40	M	Diploma	Assistant nursing officer
S 8	28	F	Diploma	Clinical officer
S 9	20	F	Secondary	Young people and adolescent peer supporter
S 10	32	M	Certificate	Counselor
S 11	45	F	Diploma	Nursing officer
S 12	32	F	Certificate	Enrolled nurse
S 13	45	F	Completed primary	Community linkage facilitator
S 14	27	F	Diploma	Nursing officer
S 15	30	M	Certificate	Enrolled nurse

### 3.2 Facilitators and barriers to ART adherence following IAC among HIV-positive clients

**Figure 2:**
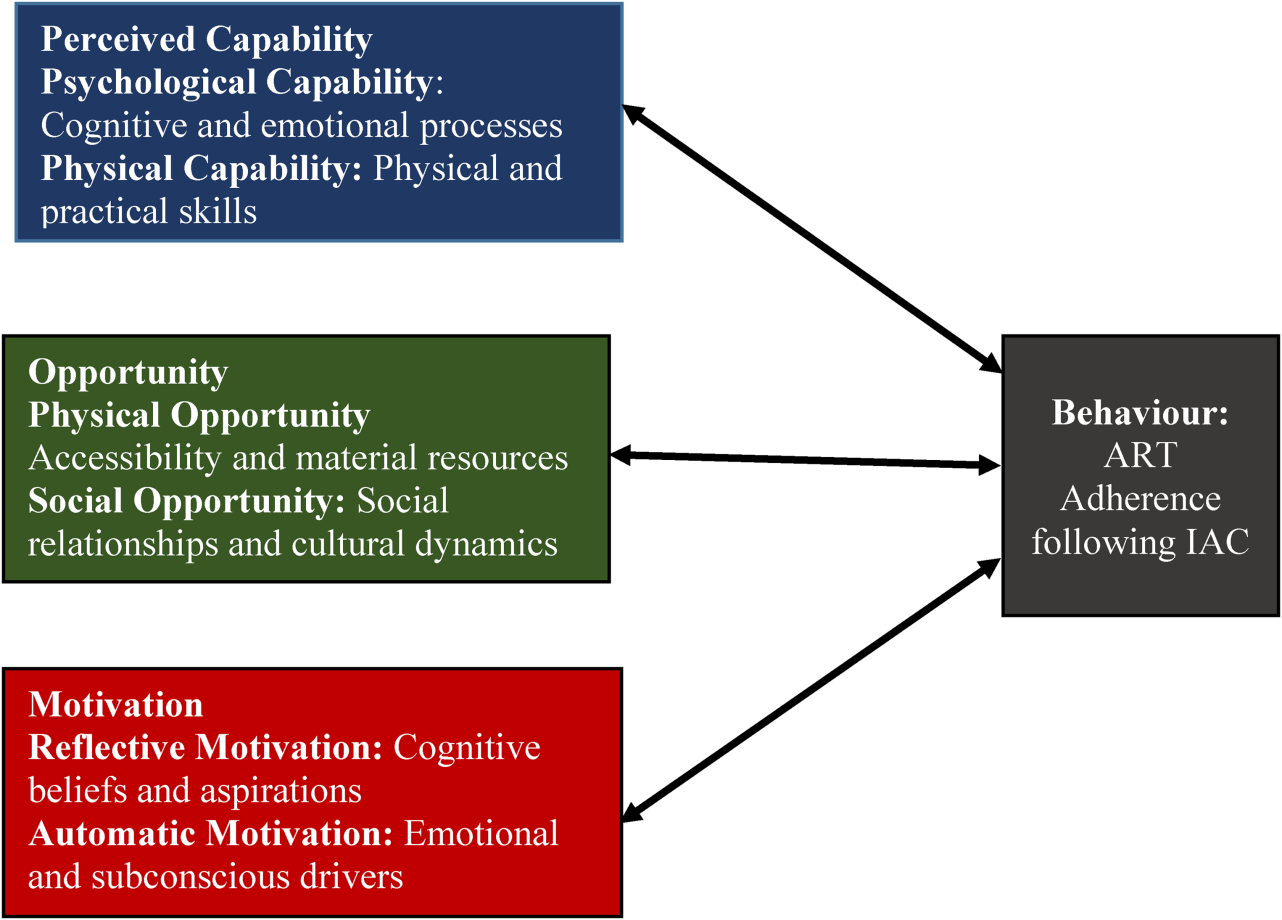
Health workers’ perspectives on the facilitators and barriers to ART adherence following IAC.

**Figure 3:**
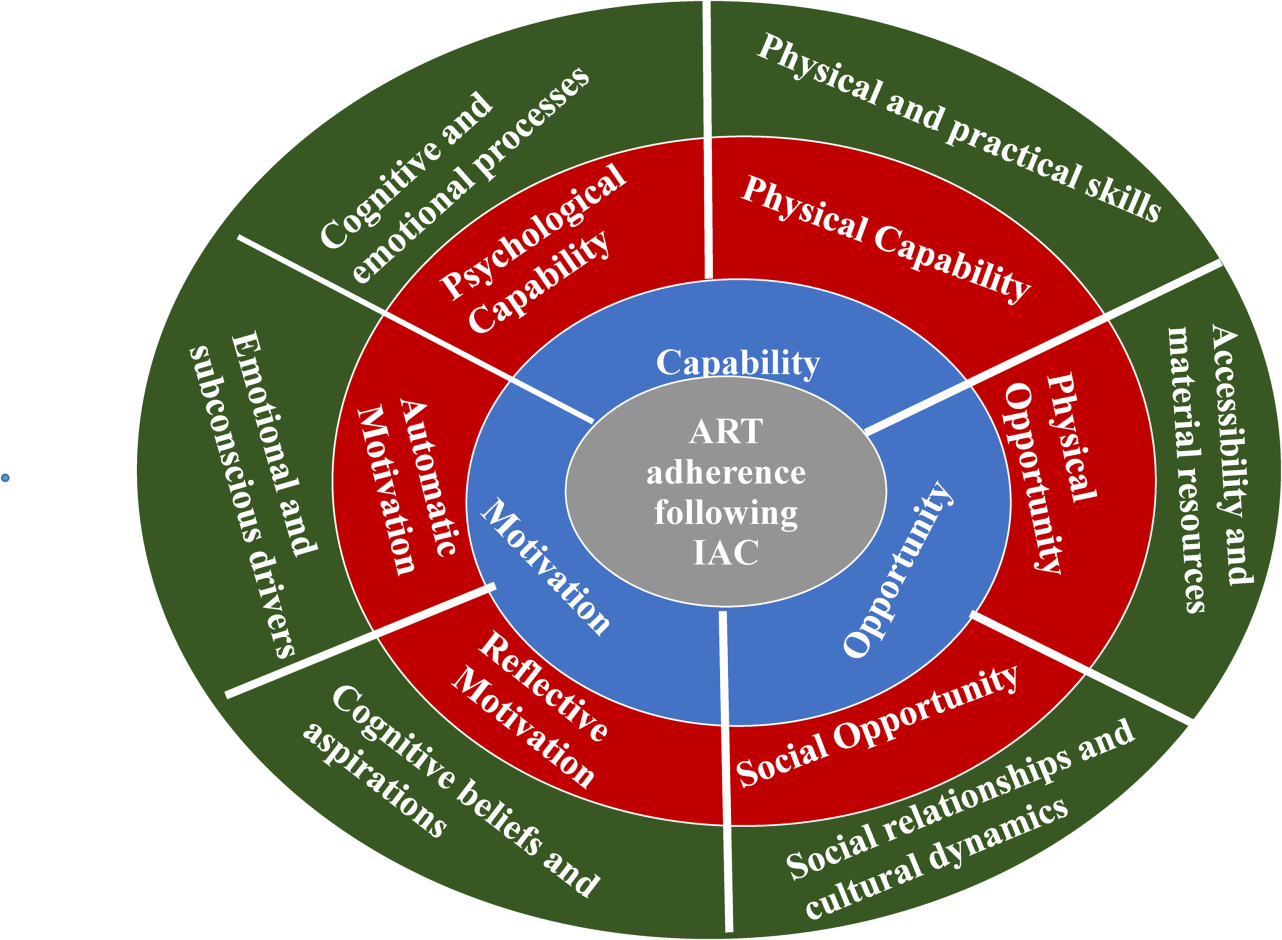
Health workers’ perspectives on the facilitators and barriers to ART adherence following IAC incorporated into the COM-B framework.

The facilitators and barriers to ART adherence following IAC were grouped into the four components of the COM-B theoretical framework, namely, capability, opportunity, motivation, and the desired Behavior (Figures 2, 3). The analyzed qualitative data were used to create high-level themes which are reported as key results under each component of the COM-B framework. The key results are dual, meaning they stand as either a facilitator or a barrier depending on the lenses of interpretation. For instance, when the results are considered from a positive angle, they become facilitators, and when considered from a negative angle, they become barriers as will be explained.

### 3.3 Perceived facilitators and barriers to ART adherence following IAC

The facilitators and barriers to ART adherence following IAC in this study were referred to as perceived facilitators and perceived barriers, as they reflect the perspectives of the healthcare workers rather than those of the PLWH themselves. These were arranged into the three domains of the COM-B framework: capability (C), opportunity (O), and motivation. Each of these domains was further divided into two subdivisions, and the high-level themes reported in this study were generated, with one theme per subdivision of the three domains. The desired Behavior (B) in this study is ART adherence following IAC.

### 3.4 Perceived capability (C)

#### 3.4.1 Psychological capability: cognitive and emotional processes

Cognitive and emotional processes emerged as a key theme in the analysis of the capability component of the COM-B framework. These processes involve the mental and emotional mechanisms that influence ART adherence, such as knowledge, memory, attention, decision-making, and behavior regulation.

The healthcare workers perceived that awareness or ignorance about viral multiplication could be a facilitator or a barrier to ART adherence following IAC. They explained that some clients do not fully understand why they are advised to adhere to ART medication, which hinders their adherence, whereas those who recognize its importance in preventing viral multiplication are more likely to adhere following IAC.

Most of the clients are ignorant on viral multiplication. They don't know that when you don't take your ARVs at the right time and the right dose as prescribed by the medical personnel. It breaks up the link of reaction of the drug and makes the virus to multiply, and I think that's why most of them take time to suppress. (Participant 2)

The health workers also perceived that some clients have misconceptions about ART medication, such as assuming that taking the drug requires eating a lot of food and having a lot of energy. As a result, they fail to take their medication if they have not yet eaten. However, those who understand that ART does not require eating food first—though good nutrition is recommended—tend to adhere to their ART better following IAC.

Some people believe that their tablets require a lot of energy and therefore they tend to avoid when there is not enough food to eat. (Participant 4)

Knowledge of ART was perceived as a facilitator and a barrier to ART adherence in different contexts depending on the angle of consideration. For instance, the health workers perceived that the PLWH who know the importance of time management in taking their drugs adhere better than those who do not understand. On the other hand, they also perceived that when the workers are well equipped with the knowledge about ART and offer quality IAC, it facilitates ART adherence following IAC compared to when the health worker is not well equipped.

Most of them appreciate the knowledge they get from us during IAC. For example, maintaining the minute and hour that they are supposed to take their drugs. Most of them complain that they have not been aware of the importance of maintaining the minutes …. (Participant 6)

With the health worker, once you are not attached to the ART clinic, then you will not have enough knowledge to handle these clients, or even when you are attached and you don't have the desire to learn and love these clients, you will fail to handle these clients and this will make them fear and definitely, they will not adhere to their medication …. (Participant 8)

Furthermore, it was perceived that PLWH who have learned how to manage their ART medication even when going on long journeys tend to adhere better than those who have not yet learned. This means that adopting the behavior of carrying along enough quantity of ART medication when traveling could facilitate adherence to ART following IAC.

When handling these clients, especially those who travel more often, we usually advise them to always pack some tablets in a suitable container to cover the duration that they will take during the journey, so that they don't miss on their drug. To me I think that most of the drivers and other travelers have adopted to this and it has made their adherence to the drugs high while in this program of IAC. (Participant 12)

It was also perceived that the ability to cope with stigma is a facilitator and the inability to cope with HIV-related stigma is a barrier to ART adherence following IAC. The health workers perceived that PLWH who can cope with stigma adhere better to ART compared with those who cannot cope with stigma. The health workers also shared that the stigma manifests at an individual level but also the community level.

I think most of the clients deny their drugs because of the problems that they have, example, stigma. (Participant 3)

… sometimes, stigma in the community also affect their adherence. Most children are non-suppressed, and most of the adolescence because they fear stigma from their colleagues and deny use of drugs, (Participant 4).

Also, when you go for; say a burial of a person who died of HIV/AIDS, people will preach against it and you find that a client who is positively living with HIV will not feel good because they will develop a mind that; ‘if you have HIV, you die’, and a person will always not have hope in life, hence making them not to take their drugs well (Participant 7).

When one is worried about stigma, it becomes even more difficult for one to disclose his/her HIV status to anyone. It was also perceived by the health workers that the ability to disclose HIV status is a facilitator whereas the inability to disclose the HIV status is a barrier to ART adherence following IAC. The health workers also shared that some parents of HIV-positive children do not inform their children about their HIV status which remains a barrier to ART adherence since these children fail to find a justification to adhere to the ART medication.

Others also have a problem of self-disclosure especially to people who are close to them. So, drug taking becomes a challenge. (Participant 3)

Some children also have problem of disclosure, i.e., they have not been guided that they are sick and reasons why they must adhere to ART. Such children end up not suppressing. So, these children do not know the reasons why are supposed to adhere to ART and they have no information about being HIV-positive. Therefore, there is need to disclose to these children. (Participant 4)

The health workers also perceived that the ability to remember the skills learned during the IAC sessions is a facilitator whereas the inability to remember is a barrier to ART adherence following IAC. It was narrated that some clients may forget the time of taking drugs while others may even forget the appointment date for drug refills; thus, they fail to adhere to ART. This was perceived as a barrier because some of them may fail to remember the techniques taught during IAC, e.g., using a caregiver to remind them, notebooks, radio news time, and alarms as reminder mechanisms to enhance ART adherence. The health workers also narrated that they perceive being attentive during IAC as a facilitator whereas being absent-minded during IAC is a barrier to ART adherence following IAC since the inattentive client may not remember all that has been discussed during the IAC session.

Through IAC, clients have learned how to take their ART medication well as prescribed by the clinicians, and during the session, clients who reported forgetfulness on the time to take medication and appointment date for drug refill learn how to use calendar and notebooks to remind them. Meanwhile, those who can use calendar and notebook, we advised them to identify a trusted caregiver at home to keep reminding them when to take their medication and return to the facility for their drug refill. (Participant 14)

People who do not take their time to listen to our guidance and counseling may lack such knowledge or skills and abilities. (Participant 6).

For those who have not suppressed, we encourage them to buy a radio that they can use to time themselves especially when it is time to take medication. (Participant 2)

The factors that inform the decision process of the clients could influence ART adherence in one way or the other. It was perceived by the healthcare worker that the ability to follow medical advice by the clients is a facilitator and the hesitancy to follow medical advice by the clients is a barrier to ART adherence following IAC. Some of these hesitant clients could be in denial, while others could be having some unaddressed myths and misconceptions which when addressed could lead to ART adherence following IAC.

However, there are some clients who do not listen to what the medical personnel tell them to do, and this has made work very hard, such are the people who don't suppress and if they are to, then they take long to suppress. (Participant 12)

Some of these myths and misconceptions could be implanted by cultural ideologists or religion-based ideologists who may ill advise the PLWH not to take ART medication because “God has healed them already.” Therefore, the health workers perceived unfavorable religious teachings and beliefs as a barrier to and favorable religious teachings and beliefs as a facilitator to ART adherence following IAC.

There is also a conflict of ideology, where people who believe in the church do claim that it is God who heals and they do not take up treatment seriously, and this sometimes lead to death. Especially the non-suppressed, they always go to the church and never come back to the facility for more medical care. (Participant 5)

It was perceived that the use of ART medication as prescribed only is a facilitator whereas unkempt behavior like mixing ARVs with herbal medicines or giving ARVs to the pigs was perceived as a barrier to ART adherence following IAC. The health workers reported that some PLWH give their ARVs to pigs or chickens for fattening while they miss taking their drugs on certain days. On the other hand, mixing ARVs with herbal medicines may result in reactions that may neutralize the effect of ARVs, resulting in no evidence of ART adherence as determined by viral load measurements.

Some of the clients on ART mix taking of ARVs with traditional drugs (herbs) which then hinder the functioning of the ARVs, and to them, they think that it is ARVs which is making them not to suppress. (Participant 2)

Misuse of ARVs (e.g., giving them to chickens or pigs) may lead to poor adherence to ART since some clients do it. (Participant 2)

These findings suggest that cognitive and emotional processes significantly shape ART adherence; thus, ART adherence and IAC-related interventions should address both knowledge gaps and emotional barriers among PLWH if optimal ART adherence is to be realized.

#### 3.4.2 Physical capability: physical and practical skills

Physical and practical skills focus on the key skills and proficiencies acquired through practice that may enable or deter one from adhering to ART. The presence of these skills and proficiencies was perceived as a facilitator whereas the absence of these skills and proficiencies was perceived as a barrier to ART adherence following IAC. The healthcare workers perceived that mastering the art of taking ARVs is a facilitator whereas the absence of the art of taking ARVs is a barrier to ART adherence following IAC. They suggested that the PLWH have to get used to taking the drugs daily and know how to take them easily without getting choked, as this can be instrumental in maintaining ART adherence.

We also ask them to demonstrate how to take the ARVs, which also develops their skills and ability which makes them adhere to the drugs. (Participant 11).

It was also perceived that having food mobilization and income-generating skill or activity is a facilitator whereas lack of food mobilization skills and income-generating activity or skill is a barrier to ART adherence following IAC. For example, they narrated that those who have learned how to grow vegetables adhere better to ART than those who have not. They narrated that clients who grow vegetables use them as food but also as a source of income to meet some of the basic needs to allow them to adhere to ART properly.

…you find that most of our clients are advised to plant greens and vegetables around their homes which helps to improve on appetite and some of which are sold on small scale. This helps meet their small-scale economic basic needs that enable them to take their drugs well. (Participant 10)

While at the facility here, we encourage the clients to plant vegetables around their homes so that food is not a problem and most of the clients enrolled in this facility grow vegetables around their homes. This has made adherence high since also, at the facility here, we advise the clients to eat well and eat a balanced diet. (Participant 15)

In the same way, those who can make money adhere better than those who cannot make money since they will have the ability to meet their basic needs and those of the family members, their self-esteem will be raised, and they will have hope for life again. These money making skills are taught to the clients during the IAC sessions. The healthcare workers also perceived that those with better socioeconomic status receive better social support from the family members since they are able to meet the needs of the family. The social support is perceived as a facilitator to ART adherence whereas lack of social support is perceived as a barrier to ART adherence following IAC; this factor is cross-cutting.

Through IAC, clients who had given up in life acquired financial skills on how to make and save money through money-generating activities like selling cassava, chips, roasting maize, making mats, and this has improved their economic status and self-esteem in the community and as well as providing basic needs of their children. (Participant 14)

… if a person earn money on a daily basis, the person will be so sure that the drug will always work well. You know you have money, and you can feed yourself well. If they say you need family support, people will always be there for you since you also support them. So, you will see that such a person will adhere to the drugs well. (Participant 7)

Social support was identified as a cross-cutting facilitator. Patients with better socioeconomic status received stronger family support, which further enhanced adherence.

### 3.5 Opportunity (O)

#### 3.5.1 Physical opportunity: accessibility and material resources

This opportunity component of the COM-B framework will report results that align with the environmental context and resources that can influence ART adherence following IAC. We found that accessibility to the healthcare facility or support and individual material resources was perceived as key to ART adherence despite IAC.

The healthcare workers perceived that those who have to travel long distances to reach the health facility for drug refills face a lot of challenges in adhering to ART due to the demotivation they get from long-distance travel. Sometimes, they may use a phone call to reach a facility if they encounter any problems with their medication. However, they may not receive comprehensive support due to the limitations of phone interactions with healthcare workers. Those who can easily reach the health facility due to proximity are favored and show better adherence to ART following IAC.

Some of the things that makes the clients not to adhere to ART will in IAC is long distance from where they stay to the facility. Here a case in point is some of our clients come from Alebtong district (far from the health facility) for the ART service here. Sometimes, if a client does not have the means of transport to the facility, the client will be disadvantaged leading to low adherence. (Participant 3)

Long distance kills the motivation of the PLWH because a client/patient may/will have to walk over a long distance to get to the health facility of even if he/she is not feeling well. (Participant 10)

It was also perceived that the presence of food is a facilitator and lack of food is a barrier to ART adherence following IAC since some clients fail to take drugs on an empty stomach. This is similar for both adults and children as evidenced by the health workers sharing a case where a client reported that her child living with HIV refused to take food on an empty stomach. This means that if there is enough food for such a person to eat, the person will take drugs as recommended and hence ART adherence will be good.

However, it is very tricky since the mother always says ‘my child doesn't want to take drugs since there is nothing to eat’. Even the child says, ‘I can only take drugs if you give me food,’ so, in that case you can't do much. (Participant 1)

Some clients lack food or money to buy food, they fear taking drug on an empty stomach, and they end up defaulting their treatment. This results to high viral load. (Participant 14)

The health workers perceived that the presence of a sufficient number of staff in the ART clinic is a big facilitator to ART adherence following IAC as the IAC team would offer high-quality counseling during IAC and IAC would achieve its full potential. However, they also perceive that understaffing is a big barrier to ART adherence following IAC since the few staff available are overwhelmed with work due to the high patient volume which affects the quality of IAC given; hence, the clients remain inadequately empowered to adhere to ART resulting into poor adherence following IAC.

There are some few counselors who are trained to handle clients here. These counselors are usually overwhelmed with work out of the few, so it is difficult to be effective in the IAC process. (Participant 15)

The healthcare workers perceive that when their salaries are paid on time, they can meet their basic needs and perform their duties diligently. However, when their salaries are delayed, they become frustrated by their numerous challenges and inability to meet their basic needs. This causes psychological distress, which impacts their ability to offer high-quality IAC to clients. As a result, clients may feel less empowered to adhere to ART, leading to poor adherence following IAC.

Now, when we have stayed like for 4 months without salary, the information that I will give to the client may not be very clear, since as you are passing the information, there could be some other things at home that requires money like your kid has been sent home for school fees. Once you're doing IAC and you think of your kid being at home when you are doing work which you are not being paid for to support the family, you get affected psychologically and you will not perform to the expectation. To me, I feel that both parties should have peaceful mind so that information can flow and adherence can take effect. (Participant 11)

It was also perceived by our study participants that those who possess property tend to adhere more to ART than those who do not. They explained that misfortunes like theft of the property of PLWH negatively affect their adherence to ART even if they have had IAC since they lose interest in being alive and wish to die, thus failing to take the drugs. The healthcare workers perceived that if one is in possession of properties, he/she is motivated to continue taking the drugs well and not to die.

At times, the community in which the client lives influence them negatively to adhere to ART, because when the community members mistreat the client on ART and while in IAC by maybe, stealing their property, such client will be discouraged in life and will feel like he/she should die. Therefore, he or she will abandon ART, and this will lead to low adherence. (Participant 15)

The quality of care offered to the clients when they come to the ART clinic was also perceived as a facilitator whereas poor-quality service was perceived as a barrier to ART adherence following IAC. The study participants perceived that reducing the waiting time for the clients when they come to the ART clinic keeps them motivated to adhere to ART. They narrated that quick service provision makes the clients happy about the services, thus making them pay attention to the IAC sessions and gain the tools they need to adhere to ART, whereas long waiting hours in the clinic do the opposite.

I think that some of them get motivated because of the way we handle them, like when they come to the facility here, we serve them quickly so that they can go back home and do other domestic work. (Participant 14)

Living in an organized and less congested environment was perceived as a facilitator, whereas living in a congested and very busy environment was perceived as a barrier to ART adherence following IAC. The health workers explained that people living in congested places like markets and schools may either be too busy and forget to take drugs or may fail to take drugs in public due to fear of stigma.

For people who live in the market and busy trading center, this kind of environment affects them negatively to ART adherence. Most of these people will tend to concentrate on business and sometimes forget taking of their medicine or others do value their business than their life. (Participant 2)

A congested environment like the school reduces adherence since most people fear to expose their status to other. (Participant 3)

The presence of fully functional community drug distribution points (CDDPs) was also perceived as a big facilitator to ART adherence as drugs are taken closer to the clients, the problem of long-distance travel is minimized, and also stigma is minimized. The health workers perceived that when CDDPs are interrupted in any way, the established system of drug refill for the clients is destroyed, thus affecting availability of the ART medication and subsequently negatively affecting ART adherence.

…as a facility, we also implemented government program of taking the services near to our clients where we take to them the drugs to their respective villages, and this has increased/promoted adherence among our clients. (Participant 3).

It was perceived that those with a living sexual partner tend to adhere better than those who have lost a sexual partner. The healthcare workers perceived that the loss of a sexual partner who is also living with HIV tends to bring a negative feeling on the remaining partner who may worry about their death at any time despite taking drugs. This emotional burden can lead to failure in taking the drugs, resulting in poor adherence.

…if it happens that also they are concordant positive and the other partner dies, this remaining person will also feel that at any time he/she may also pass away even if he/she continues taking drugs, and so they become relaxed in drug taking making their ART adherence low. (Participant 7)

Addressing barriers such as distance, food insecurity, and staffing shortages can improve ART adherence. Policies that expand CDDPs and improve healthcare worker welfare are recommended to enhance the IAC process.

#### 3.5.2 Social opportunity: social relationships and cultural dynamics

This focuses on the social influences on ART adherence like social pressures, norms, conformity, and social comparisons. The study participants in this study perceived that some of the enhancing social relationships and cultural dynamics are facilitators, whereas those deleterious ones are barriers to ART adherence following IAC. The healthcare workers reported that some of the clients especially widows or widowers are mistreated by their relatives or other family members which makes them stay in a stressful situation that hinders their ability to adhere to the ART medication. This means that these mistreatments could be informed by some of the cultural norms and expectations which may not be in alignment with the desires of the person being mistreated.

Some clients especially widows are also being disturbed with home issues hence resulting into stress as well as missing drugs. (Participant 2)

The availability of peer and social support for PLWH was perceived as a facilitator, whereas the absence of peer and social support was perceived as a barrier to ART adherence following IAC. The health workers perceived that clients who spend most of their time with PLWH show better adherence to ART compared with their counterparts, whereas clients with a supportive caregiver adhere better than those with non-supportive caregivers.

Clients who live with fellow clients tend to adhere to ART compared to clients who prefer to stay with people who are negative. (Participant 2)

Another force that derails these clients' ability to take ART medicine is non-supportive caregivers such that if they fall sick, the caregiver will not mind about them. (Participant 3)

In the same way, the healthcare workers perceived that spending time with fellow PLWH gives the person on ART the opportunity to learn from the good stories and testimonies, which facilitates ART adherence following IAC. On the other hand, spending time with those who are not living with HIV and are very negative about PLWH may lead to a feeling of being stigmatized, which may eventually lead to other psychological consequences like stress and depression that hinder ART adherence following IAC. The health workers narrated that an environment which is favorable for ART adherence will act as a facilitator whereas an environment which is not favorable will act as a barrier.

Like for example when the environment is favorable for the client, it influences the adherence positively for example when a client lives within the peers where they are usually free to share ideas with the client, they can be of help. But in an event that a client lives in an environment which is hostile for them, they are exposed to stigma, which makes them depressed, and this will eventually lower their adherence to ART medication …. (Participant 9)

It was also perceived that a favorable home environment with good family cohesion and no domestic violence is a big facilitator whereas an unfavorable home environment with numerous family wrangles and domestic violence is a barrier to ART adherence following IAC. This is because violence may lead to displacement, and the displaced person may not carry the drugs along or may get disconnected from the ART clinic where he/she receives ART services, hence affecting ART adherence despite IAC. Furthermore, it was perceived that handling clients well and with respect by the healthcare workers is a facilitator whereas poor handling is a barrier to ART adherence as those handled poorly may not come back for drug refills.

Sometimes the clients may be facing domestic violence such that, when they fight and they part ways, such a client is displaced away from his/her medication. This may lead to skipping of the ARVs by the client who is under IAC and hence making this client to have low adherence. (Participant 12).

The way ‘we’ the medical practitioners handle them at times makes the client not to come to the facility especially for drug refill. (Participant 14)

There are some staff who are tough and rude, and they transfer their aggression to the clients. When these clients meet them, they may boycott coming to the facility for support services and IAC; in other words, they become lost clients, and this makes them not to adhere to the ART. (Participant 12)

The healthcare workers also perceived that those who have a single sexual partner tend to adhere better than those who engage themselves in multiple sexual relations. They explained that those who have many sexual partners may contract other HIV-related comorbidities that may negatively affect their ability to adhere to ART even after IAC.

Also, some clients get engaged in sexual intercourse with many people outside marriage. This makes them weak or they sometimes end up contracting a resistant variant or even they contract other diseases that affects their immunity and they remain non-suppressed. (Participant 3)

It was also perceived that reaching out to the community to create awareness about HIV, ART, and ART adherence is a facilitator as those in the community are equipped with knowledge on how to handle those who are living with HIV and those on IAC. The health workers also suggested that these community activities could be used to demystify some of the myths and misconceptions about ART medication among the community members that may act as barriers to ART adherence. They shared a case in point where some of the PLWH think the health workers make money when they take ART medication.

Usually, we organize a sensitization program with the community on how to take drugs and treat the fellow, who has HIV in intensive adherence counseling program, and even the kind of support to give them, which also makes them strong to come for the IAC program…. (Participant 15)

Some people have a negative thought that we are making money out of them that is why they don't come. (Participant 11)

### 3.6 Motivation (M)

#### 3.6.1 Reflective motivation: cognitive beliefs and aspirations

Reflective motivation involves the cognitive beliefs and aspirations that shape individuals' decisions and actions, particularly their intentions, future goals, and identity. These cognitive aspects influence the way people perceive the consequences of their actions, such as taking or not taking ART medication. In this study, we, the healthcare workers, perceived that PLWH who have a clear sense of future goals and a desire to stay alive tend to adhere better to ART.

…, when they are counseled, they feel free and happy again because. When you talk to some of them for long, they tell you that they have lost hope in life, and some of them tend not to adhere to their drugs because they want to take away their lives, but when you keep counseling them, they feel free and gain hope in life again. (Participant 10)

In the same way, the health workers perceive that the desire to stay alive is a facilitator whereas loss of interest in being alive is a barrier to ART adherence following IAC. Our study participants perceive that PLWH with resources and future plans and goals have a desire to stay alive; hence, they tend to adhere better to ART compared with those who have lost interest in life.

People who have money (rich people) use their medication prescription well since they want to enjoy the assets that they have; they never want to be attacked by other disease. (Participant 5)

Interventions focusing on reinforcing hope, future goals, and personal resources may help improve adherence. IAC and ART adherence strategies that emphasize the value of life and the benefits of staying healthy could be effective in improving ART adherence among PLHW.

#### 3.6.2 Automatic motivation: emotional and subconscious drivers

Automatic motivation refers to emotional and subconscious factors, such as emotional reinforcement, rewards, incentives, and punishments, that drive behaviors without deliberate intention. In the case of ART adherence, these emotional drivers such as the impact of stress, depression, and substance abuse can significantly influence patients’ ability to adhere to their medication regimen.

The healthcare workers perceived that PLWH who do not have life stressors that may lead to symptoms of depression adhere better to ART following IAC than those who have symptoms of depression. They also perceived that PLWH experiencing many psychosocial stressors may resort to harmful practices like excessive alcohol consumption and drug abuse, some of which may negatively impact cognitive function, leading to poor memory, which may, in turn, affect their ability to remember to take their medication, resulting in poor adherence even after IAC.

Another thing is depression which is common with adults because in our community, people know little about depression. For them, they just treat it as disease caused by overthinking. So, at the end of the day, you find that someone easily forget especially women, and for men when they are depressed, they only resort to drinking, hence making them forget. (Participant 1)

As perceived by the health workers, PLWH who do not consume alcohol or abuse drugs tend to adhere better to ART following IAC. This is because alcohol and drugs can impair cognitive function, causing individuals to forget their medication, fail to bring it with them, or miss the correct time to take it.

I think most of them have not suppressed because they spend most of their time drinking and do not return home in time to take their medicine and sometimes come back when they are drunk and forget to take the drugs…. (Participant 5)

The other one is one someone is drug addict. There are some people who are addicted to drugs like marijuana, tobacco, opium; instead of taking ART, they are so much addicted because when they inject themselves with the drugs or take the drugs. They feel high or drunk they don't think so much about taking drugs; some of them may think, ‘When you are drunk, then HIV is also drunk, so, there is no use for the ARVs.’ (Participant 7)

However, there are some of the clients who take long to adhere to their ART because of being drunkards. They don't follow the time for ARV taking, and these people resort to taking ART at their own time and at time they miss taking their drugs even when on IAC. (Participant 13).

The health workers also perceived that PLWH who accept their HIV status and make a decision to live positively adhere better to ART unlike those who remain in denial. They explained that some of these clients may not believe that they are HIV-positive and may not embrace the ART medication leading to poor adherence even after IAC.

I experienced that most patients remain in denial, and it takes them time to accept that they need to take their medication well to suppress viral load. (Participant 14)

The health workers perceived that PLWH who are not virally suppressed tend to adhere better to ART when offered quality IAC, compared to when the quality of IAC is suboptimal. They explained that using practical, easy-to-understand examples during counseling helps clients better comprehend the information, leading to better adherence. In contrast, abstract concepts during counseling are less effective. Additionally, health workers identified peer counseling as a facilitator of ART adherence, whereas lack of peer counseling was seen as a barrier.

Another thing that could have motivated them to take medicine is what we always share with them through the IAC especially on role model, what we do, and we also give example to our self that we also take medicine (ART) and we are normal. I think it has motivated them. (Participant 3)

We only build on their confidence by giving ourselves as examples, like I usually tell them that if I can take my drugs well, they can as well take their drugs well, and I also tell them how I have managed to adhere to ART. (Participant 4)

During the counseling process, avoiding negative statements by the counselor was perceived as a facilitator whereas the use of negative and judgmental statements was perceived as a barrier to ART adherence following IAC. The health workers perceived that PLWH get discouraged when they are not treated with empathy during the IAC sessions.

Our clients are discouraged by negative statements like ‘didn’t I tell you to adhere to ART’. In that, they feel useless, they feel they have made a great mistake, and they end up not taking drugs. (Participant 7)

During the counseling processes, it is perceived that discussions that bring out the benefits of ART adherence is a facilitator whereas having no such discussions is a barrier to ART adherence following IAC. During IAC, the healthcare workers motivate the PLWH to take their drugs well so that they do not have to visit the hospital frequently for drug refills since those who are suppressed are given drug refills for a longer period of time.

But we normally give the suppressed people three-month dose and the non-suppressed for one month. This is to encourage the non-suppressed to come back regularly to give us enough time to monitor these clients because when we give them for three months or more, they will forget to come back for counseling. (Participant 5)

To improve ART adherence, IAC and other ART adherence interventions could focus on addressing emotional stressors, reinforcing positive behaviors, and providing empathetic counseling. Peer counseling, as well as counseling that fosters hope, can be effective in motivating PLWH to remain committed to their treatment.

## 4 Discussion

This study aimed to explore the healthcare workers’ perspectives on the facilitators and barriers to ART adherence following intensive adherence counseling (IAC) using the Capability, Opportunity, and Motivation framework for Behavior change (COM-B framework) (18). The results of this study we discussed through the lens of multiple implementation science frameworks, including the COM-B model (18) and the Consolidated Framework for Implementation Research (CFIR) (26, 27), and other key implementation theories, such as the Theoretical Domains Framework (TDF) (28) and the Normalization Process Theory (NPT) (29). These frameworks were used to discuss the six key themes that emerged from this study, namely, cognitive and emotional processes, physical and practical skills, accessibility and material resources, social relationships and cultural dynamics, cognitive beliefs and aspirations, and emotional and subconscious drivers. The discussion synthesizes these findings and integrates them with insights from the broader literature to provide a comprehensive understanding of ART adherence following IAC and to inform future targeted adherence interventions.

### 4.1 Perceived capability

#### 4.1.1 Psychological capability: cognitive and emotional processes

The study participants perceived that cognitive and emotional processes are critical in shaping ART adherence since having knowledge about ART and ART adherence, the ability to manage stigma, and the ability to remember and apply them well can facilitate ART adherence. The result of this study is consistent with that of a study in Nepal and another study in India which also found that having misconceptions about ART medication is a barrier to ART adherence whereas having knowledge of ART medication and how to adhere to the medication facilitates adherence (30, 31). Similarly, a systematic review of six studies from 2015 to 2021 on the factors affecting adherence to ART by PLWH In Nigeria found that a gap in knowledge about HIV was a barrier to ART adherence among the study participants (32). From an implementation science perspective, this is reflected in the TDF, which identifies beliefs about capabilities and emotion regulation as key domains influencing behavior (28). The healthcare workers can focus on ensuring the PLWH on IAC capture the knowledge about ART, are clear from misconceptions about ART, and are well equipped to adhere to their medication. Integration of continuous knowledge support and management into ART adherence interventions is critical, as NPT emphasizes the importance of collective action (e.g., a joint work between patients, caretakers, and healthcare providers) to ensure effective treatment management (29). Emotional barriers could be addressed through mental health-related interventions that can improve adherence outcomes by reducing distress related to stigma and enhancing patients' capability to manage their treatment regimen.

#### 4.1.2 Physical capability: physical and practical skills

In terms of physical capability, participants perceived that PLWH with skills to manage their medication schedules and handle side effects have higher adherence to ART compared to those who do not. This study further highlighted the key challenges involved in managing ART schedules like when traveling long distance and lack of reminder alerts, among others. The result of our study aligns with that of a qualitative study conducted among people who inject drugs which found that taking pills and attending appointments together for couples living with HIV, using reminders to take pills, and providing babysitting to enable attendance at doctor appointments were facilitators to ART adherence among the study participants (33). These facilitators fit perfectly into the broader physical and practical skills categorization shown by our study to be perceived to influence adherence to ART among PLWH from the healthcare workers’ perspectives. Similarly, an exploratory interview with PLWH that also used the COM-B framework found that the skills of using reminder alerts like radios and alarms, among others, to keep track of the ART schedules were facilitator to ART adherence among PLWH on IAC (12). These findings are in agreement with TDF's domain of skills, which suggests that the ability to perform a particular behavior such as taking medications consistently is fundamental in determining the success of a particular behavior, in this case, ART adherence following IAC (28). The CFIR's inner setting domain emphasizes that the organizational environment such as the availability of adequate healthcare training and medication management systems is crucial for enabling individuals to perform desired behaviors like ART adherence (26, 27). During IAC, healthcare workers should consider focusing on strengthening the skills necessary for one to adhere to ART for PLWH to optimally adhere to ART. Implementation studies on medication adherence with focus on the physical and practical skills that can enhance maximum adherence to ART could be considered.

### 4.2 Opportunity

#### 4.2.1 Physical opportunity: accessibility and material resources

The study revealed that physical opportunity, particularly access to healthcare services, medication availability, and transportation challenges, had a major impact on adherence. This is consistent with findings from a systematic review of six studies from Nigeria which found that regular clinic appointment was a facilitator to ART adherence among PLWH (32). Similarly, a most recently published systematic review of 16 studies in sub-Saharan Africa published from 2016 to 2023 found that PLWH with socioeconomic difficulties have challenges in adhering to ART medication (34). A Namibian qualitative study also showed that healthcare provider-related factors influence adherence to ART medication among older adolescents and adults living with HIV; these factors may affect accessibility to ART services (35). A systematic review of 66 studies, with 41 quantitative, 16 qualitative, and 9 mixed-methods study designs among adolescents, with a total of 53,217 participants, found that some of the barriers to ART adherence were economic and health system-based whereas PLWH access to counseling was a facilitator to ART adherence (36). In implementation science, the CFIR's outer setting domain highlights how external factors, such as economic conditions and transportation availability, influence the success of interventions (26, 27). IAC and other adherence interventions should focus on mitigating the access and material resource-related barriers and enhancing the access and material resource-related facilitators to ART adherence to achieve optimal adherence to ART medication.

#### 4.2.2 Social opportunity: social relationships and cultural dynamics

Social opportunity emerged as both a perceived facilitator and barrier to ART adherence following IAC, depending largely on the presence of family/peer support and community attitudes toward HIV. The study participants perceived that PLWH who received support from their partners or families/friends are more likely to adhere to ART, whereas those facing HIV-related stigma struggle with disclosure and secrecy. The findings of this study are similar to that of a systematic review of 33 studies conducted in 15 LMICs. This review used mixed-methods synthesis and found that cultural context, religious beliefs, and social norms reinforced or undermined household support for ART adherence and that stigma affected disclosure, generated secrecy around giving medication, and impeded access to support for PLWH from the community (37). Similarly, another systematic review of six studies in Nigeria from 2015 to 2021 found that the presence of social support from family members and friends was a facilitator of ART adherence among PLWH (32). Violent relationships have been also found to be an obstacle to ART adherence as shown by a daily diary investigation study among boys which aligns with the results of this study (38). Still in agreement with our study, a study found that social environmental factors influence adherence to ART either positively or negatively depending on the angle of the factor (39). A study in Namibia also found that ART adherence is influenced by family-, community-, and social-related factors which perfectively fit into the social relationships and cultural dynamics theme of our study (35). Compared with some of the implementation science theories, the findings of our study echo the NPT's concept of collective action, where social and institutional support is key to the normalization of behaviors such as ART adherence following IAC (29). Similarly, the results of our study also align with the TDF which states that social influences are critical in shaping behaviors like adherence to ART following IAC (28). Family members and peers can act as motivators for ART adherence to optimize adherence among PLWH. When social norms reinforce stigma or discourage disclosure, adherence to ART can be undermined as reflected by our study. To address this, interventions should aim to reduce stigma through continuous and sustainable community engagement and peer support networks.

### 4.3 Motivation

#### 4.3.1 Reflective motivation: cognitive beliefs and aspirations

Participants who had strong cognitive beliefs about the benefits of ART, including disease prevention and improved quality of life, were more motivated to adhere. The result of this study is consistent with that of a descriptive cross-sectional study conducted from June to August 2023 among 262 respondents which found that individual factors influence adherence to ART among the study participants (39). Our study therefore clearly shows that cognitive beliefs and aspirations could be the determinant of the individual factors that influence ART adherence. This finding supports the Theory of Planned Behavior, which proposes that attitudes toward health outcomes strongly influence a behavior like ART adherence following IAC (17). A qualitative study on ART adherence among young and older adults in Namibia also highlighted that patient-related factors influence whether a person will adhere to ART or not (35); we suggest that factors could be influenced by cognitive beliefs and aspirations as found by our study. The consolidated framework for implementation science's focus on individual characteristics such as self-efficacy and outcome expectancy aligns with the findings of our study too (26, 27). To enhance the reflective motivation of PLWH to adhere to ART, IAC and any other ART adherence interventions should focus on building self-efficacy and reinforcing positive beliefs about ART among the PLWH. Continuous, targeted, and goal-oriented health education campaigns that emphasize the long-term benefits of ART adherence could help shift attitudes about ART and improve the motivation of PLWH to adhere to ART.

#### 4.3.2 Automatic motivation: emotional and subconscious drivers

The emotional and subconscious drivers of ART adherence, such as fear of illness progression or emotional fatigue from lifelong treatment, were significant motivators for some participants, while others struggled with adherence due to emotional distress. This echoes a systematic review of six studies from 2015 to 2021 on the factors affecting adherence to ART by PLWH which found that those who consume alcohol were less likely to adhere to ART compared to those who did not consume alcohol (32). This could be because alcohol interferes with the normal emotional processes that may subsequently interfere with the ability to adhere to ART medication. A systematic review of eight articles comprising 6,474 participants found that having signs and symptoms of depression was a risk factor for non-adherence to ART among the study participants (40). Similarly, a cohort study among adolescents and adults in South Africa found that those who had a diagnosis of any mental illness had unfavorable ART adherence patterns unlike those who did not have such a diagnosis (41). This could be because mental illnesses alter the emotional and subconscious functions within an individual, hence affecting adherence to ART following IAC. On the other hand, a systematic review of 16 studies in sub-Saharan Africa found that lack of motivation is a barrier to ART adherence following IAC (34). This result does not show an action-oriented barrier to ART adherence since the factors underlying the lack of motivation were not clearly defined. Our study brings in new evidence that lack of motivation to adhere to ART could be driven by emotional and subconscious drivers which we have fully explained in the results section of this study. From an NPT's perspective, emotional reactions to ART treatment can influence its normalization within the daily life of a person (29). Psychosocial support is essential to reduce the emotional burden of living with HIV, and IAC and other targeted interventions for ART adherence should focus on the emotional well-being of PLWH to improve automatic motivation for optimal ART adherence.

## 5 Limitations of the study

The use of the COM-B framework for behavior change in this study and the implantation of science theories in discussing the results enabled the researchers to demonstrate how the results of this study can be used in the wider implementation science field to inform adherence interventions. This study focused exclusively on the perspectives of healthcare workers (HCWs), potentially overlooking firsthand experiences of people living with HIV (PLWH). However, this was intentional, as an existing study has already explored the perspectives of PLWH on barriers and facilitators to a successful IAC (42). Our study aimed to complement that work by providing insights from HCWs, who play a critical role in implementing IAC to enhance ART adherence among PLWH who are virally non-suppressed. This study also richly explored the available literature on ART adherence and comprehensively synthesized how the available evidence can be used collectively to optimize adherence to ART among PLWH.

This study did not include the perspectives from other stakeholders such as policymakers on the facilitators and barriers to ART adherence following IAC, as their input could have offered a broader understanding of systemic or policy-related facilitators and barriers to ART adherence following IAC. However, policymakers can use the evidence generated by this study and other studies to inform their policy decisions on the care for PLWH. While purposive sampling ensured the inclusion of participants with relevant experience, HCWs from different contexts like the lower-level facilities were not represented; however, this may not affect the generalizability of the results since some of our study participants were also working in such low-level facilities at the time of the study and worked across multiple health facilities. Because of the qualitative approach used in this study, our results may not be generalizable to a wider context although it can be transferrable to similar contexts. However, this limitation was turned into a strength by comparing the results of this study with those of other high-level studies such as systematic reviews and implementation science theories, all of which are in agreement with the results of this study.

## 6 Conclusion

This study provides new evidence for a better and clearer understanding of the facilitators and barriers to ART adherence following IAC using a high-level categorization with the COM-B framework for behavior change while explaining how these facilitators and barriers fit into some of the existing implementation science theories, such as the consolidated framework for implementation science, theoretical domains framework, and the normalization process theory. This study therefore offers a multidimensional view of how the factors influencing ART adherence following IAC can be optimized for the best outcome of the intensive adherence counseling program.

These findings suggest that optimizing cognitive and emotional processes, physical and practical skills, accessibility and material resources, social relationships and cultural dynamics, cognitive beliefs and aspirations, and emotional and subconscious drivers during IAC and any ART adherence-related intervention could yield the best level of ART adherence among PLWH who are virally non-suppressed and on ART. IAC and ART adherence interventions should incorporate multi-level strategies, including community engagement, healthcare provider training and refresher training, and routine mental health screening and support for PLWH, to improve ART adherence and health outcomes for PLWH.

## Data Availability

The raw data supporting the conclusions of this article will be made available by the authors, without undue reservation.
