# Supplementary material for: Perspectives of health workers on the facilitators and barriers to antiretroviral therapy adherence following intensive adherence counseling in Northern Uganda
Source: Front Health Serv. 2025 Jan 28;5:1387823. doi: 10.3389/frhs.2025.1387823 (PMC11810930; doi:10.3389/frhs.2025.1387823)
Supplement: Supplementary file 1 [file Supplementaryfile1.docx]

| **Title** | Perspectives of health workers on the facilitators and barriers to antiretroviral therapy adherence following intensive adherence counselling in northern Uganda |
| --- | --- |
| **Respondent ID** |  |
| **Date of the interview** |  |
| **Introduction** | Introduce yourself, build rapport with the participants, and ensure the informed consent form is signed |
| **Socio-demographic characteristics** | Please tell me more about yourself  (Name, age, sex, cadre, role at the facility, years of work experience) |
| **Discussion guide** | **Probes** |
| **ART adherence** | 1. What do you know about IAC? 2. How is the attendance to IAC sessions in your facility? 3. What are your experiences during IAC sessions? 4. How is ART adherence after enrolling in IAC among clients here? |
| **Capability** | 1. What knowledge, skills, abilities, or proficiencies acquired through the IAC program facilitate adherence to ART? 2. What gaps in knowledge, skills, and abilities hinder adherence to Antiretroviral therapy during intensive adherence counselling? |
| **Opportunities** | 7). How does environment or resources influence adherence to ART even during intensive adherence counselling?  8) How do social influences (social pressure, norms, conformity, and social comparison) influence ART adherence while in the IAC program? |
| **Motivation** | 9) What motivates clients to adhere to their medicine while in the IAC program?  10) What kills the clients’ motivation to adhere to medicine while in the IAC program? |
| **The end of the interview** | Could there be anything you would like to add?  I will look into the information you have provided and write a report that will be submitted to an authentic peer-reviewed journal for a possible publication. If you need a copy, I will be willing to send you one when the time comes.  Thank you participating in this study. |
